# Drug utilization patterns and healthcare resource use and costs in patients with neurogenic bladder in the United Kingdom: A retrospective primary care database study

**DOI:** 10.1002/nau.23981

**Published:** 2019-03-28

**Authors:** Ashley Jaggi, Jameel Nazir, Francis Fatoye, Emad Siddiqui, Nurul Choudhury, Ramzi Argoubi, Mahmood Ali, Dirk de Ridder, Marcus J. Drake

**Affiliations:** ^1^ Department of Health Professions Manchester Metropolitan University Manchester United Kingdom; ^2^ Health Economics and Outcomes Research Astellas Pharma Europe Ltd Chertsey United Kingdom; ^3^ Health Economics and Outcomes Research Creativ‐Ceutical SARL Les Berges du lac Tunisia; ^4^ Department of Urology, Organ Systems, Development and Regeneration University of Leuven Leuven Belgium; ^5^ School of Clinical Sciences Translational Health Sciences, Bristol Medical School, University of Bristol Bristol United Kingdom

**Keywords:** cholinergic antagonists, comorbidity, healthcare costs, healthcare resources, neurogenic, retrospective studies, urinary bladder

## Abstract

**Aim:**

To characterize patients with neurogenic bladder (NGB), their treatment patterns, healthcare resource utilization, and associated costs based on records from a primary care database in the United Kingdom.

**Methods:**

This was a retrospective, descriptive, observational study of anonymized data from the Clinical Practice Research Datalink and Hospital Episode Statistics databases (selection period, 1 January 2004 to 31 December 2016). Adults with a definitive or probable diagnosis of NGB and ≥1 referral to a urologist were included.

**Results:**

The study cohort included 3913 patients with definitive (n = 363) or probable (n = 3550) NGB. Patients had a mean of 8.6 (standard deviation [SD], 7.6) comorbidities, and mean Anticholinergic Cognitive Burden Scale score of 6.6 (SD, 5.9). During 12 months’ follow‐up, urinary tract infection (UTI) and urinary incontinence were the most common complications. Most patients (92.2%) received ≥1 prescription for an antimuscarinic agent or mirabegron, and 53.9% of patients received prescriptions for UTI‐specific antibiotics. The mean number of visits to a general practitioner for any cause was 67.7 (SD, 42.6) per individual. Almost half (46.7%) of the study cohort visited a specialist during the 12‐month follow‐up period, and 11.0% had ≥1 hospital admission. Total mean per patient costs for healthcare resource utilization was £2395.

**Conclusions:**

The burden of illness, healthcare resource needs, and associated costs among patients with NGB are considerable. Drug prescribing patterns are consistent with the symptoms and complications of NGB, although increased awareness of drugs with anticholinergic activity among prescribers may help to reduce the cumulative anticholinergic burden in this vulnerable population.

## 1 INTRODUCTION

Neurogenic bladder (NGB) is a general term used to describe lower urinary tract dysfunction secondary to neurological disease or central nervous system (CNS) injury.^1^ It is thought to affect more than 90% of patients with spina bifida and spinal cord injury, 50% to 80% of patients with multiple sclerosis,^2^ 37 to 72% of patients with Parkinson's disease, and 15% of patients with stroke.^3^

NGB has many clinical presentations, and the exact type of urinary tract dysfunction depends on the site, extent, and evolution of the neurological lesion.^4^ Urinary symptoms include frequency, urgency, and urinary incontinence. Patients may also be at risk of urinary tract infection (UTI), bladder outlet obstruction, and more serious long‐term sequelae of urosepsis and renal failure.^3^ For patients with neurological disease, lower urinary tract dysfunction may be one of the worst aspects of their condition,^4^ and symptoms are known to have a marked negative effect on quality of life.^5^, ^6^, ^7^

The goals of treatment for NGB are to restore lower urinary tract function, achieve or maintain urinary continence, protect against renal failure, improve quality of life,^8^ and minimize the risk of complications, such as UTI.^9^ Management strategies include noninvasive conservative treatments, catheters, and surgery, as well as pharmacological therapies.^4^, ^8^, ^10^ The main pharmacological treatments are antimuscarinic agents for neurogenic detrusor overactivity (NDO) and antibiotics for UTIs.^4^, ^8^

There is little information on how patients with NGB are managed in clinical practice,^1^ and no studies have been done in the United Kingdom (UK). The primary objective of this study was to characterize patients with NGB in terms of demographics, comorbidities, and complications, and to evaluate their adopted treatment patterns in terms of drug utilization. The study also assessed healthcare resource utilization and associated costs in these patients during a 12‐month follow‐up period, based on records from a primary care database in the UK. These study objectives were also explored separately according to the underlying neurological condition, as this may influence symptom presentation and severity.

## 2 PATIENTS AND METHODS

### 2.1 Study design and data sources

This was a retrospective, descriptive, observational study performed using anonymized data from the Clinical Practice Research Datalink (CPRD) GOLD and Hospital Episode Statistics (HES) databases. CPRD is a longitudinal primary care research database that collates medical records from 674 general practices across the UK, and is representative of the national population in terms of age, sex, and ethnicity.^11^ It contains information on patient demographics, prescriptions, medical history, diagnostic testing, and secondary care referrals. HES collates data on inpatient, outpatient, and accident and emergency admissions from National Health Service (NHS) hospitals in England; approximately 58% of practices within the CPRD network have consented to a linkage scheme enabling patient‐level data to be linked to other databases including HES.^11^

The study was conducted in compliance with requirements for ensuring the rights of participants in noninterventional studies.^12^

### 2.2 Study population

The study selection period was from 1 January 2004 to 31 December 2016. Adults aged ≥19 years with a definitive or probable diagnosis of NGB were included. A definitive diagnosis required ≥1 diagnosis of NGB or neuropathic bladder within the study selection period. A probable diagnosis required a diagnosis of Parkinson's disease, multiple sclerosis, spinal cord injury, or stroke within the study selection period; or a diagnosis of spina bifida within the entire CPRD database, in addition to a subsequent diagnosis of overactive bladder (OAB) and/or ≥1 prescription for an OAB medication. Patients were also required to have ≥12 months of continuous enrollment in the CPRD database before the index date (defined as the date of diagnosis of the neurological condition or, for patients with spina bifida, the date of OAB diagnosis or OAB drug prescription, whichever came first), and 12 months continuous enrollment after diagnosis of OAB or OAB drug prescription (Figure S1). All patients were required to have ≥1 referral to a urologist within 12 months before or 12 months after the index date. Patients with idiopathic OAB, a diagnosis of dementia, or those missing data for age or sex were excluded. Only patients deemed acceptable for research by the CPRD, and for whom the study period occurred during an uninterrupted period where the practice was deemed “up to standard,”^11^ were included.

### 2.3 Study objectives and endpoints

Full details and definitions of study endpoints are provided in Table S1. For the primary objectives, comorbidities were assessed using a proxy measure, that is, the number of drug classes prescribed according to British National Formulary (BNF) headers.^13^ Complications considered were UTIs, urinary incontinence, sepsis/septicemia, urinary retention, obstructive uropathy, renal failure, and hydronephrosis. Drug utilization at index date was described by prescriptions for oral OAB drugs, prescriptions for drugs with anticholinergic activity, Anticholinergic Cognitive Burden (ACB) Scale score calculated within 1 month before and after first OAB/NGB diagnosis or OAB drug prescription date (see Appendix for details),^14^, ^15^ and polypharmacy, that is, the number of substances prescribed according to BNF headers. Drug utilization during the 12‐month follow‐up period included prescriptions for oral OAB drugs, OAB drug combinations (ie, prescriptions overlapping for >30 days), α‐adrenergic antagonists or 5α‐reductase inhibitors, and UTI‐specific antibiotics.

Secondary objectives were to describe healthcare resource utilization and related costs during the 12‐month follow‐up period. Resource use was defined as all‐cause general practitioner (GP) consultations, urological investigations (urodynamics, cystoscopy, imaging), specialist visits (urologist and gynecologist), prescriptions for incontinence pads, procedures/surgical interventions (urology), and hospital visits (urology). Costs were estimated by multiplying each occurrence of resource use by unit costs derived from NHS tariffs or other UK‐specific sources^16^, ^17^, ^18^ (Table S2).

### 2.4 Data analyses

Statistical analyses were descriptive only. Analyses were performed in the overall study cohort and stratified by underlying neurological condition (Parkinson's disease, multiple sclerosis, spinal cord injury, stroke, and spina bifida), age (19‐65 vs >65 years), and sex (female vs male). A sensitivity analysis of the primary objective was performed using an alternative definition of patients with probable NGB, that is, the diagnosis of neurological condition and OAB diagnosis/OAB drug prescription could be in any order. Analyses were performed using SAS, version 9.4 (SAS Institute, Cary, NC).

### 2.5 Ethical approval

Approval for the study protocol was obtained from the CPRD Independent Scientific Advisory Committee [protocol: 17_207RMn].

## 3 RESULTS

### 3.1 Study population

**Figure 1:**
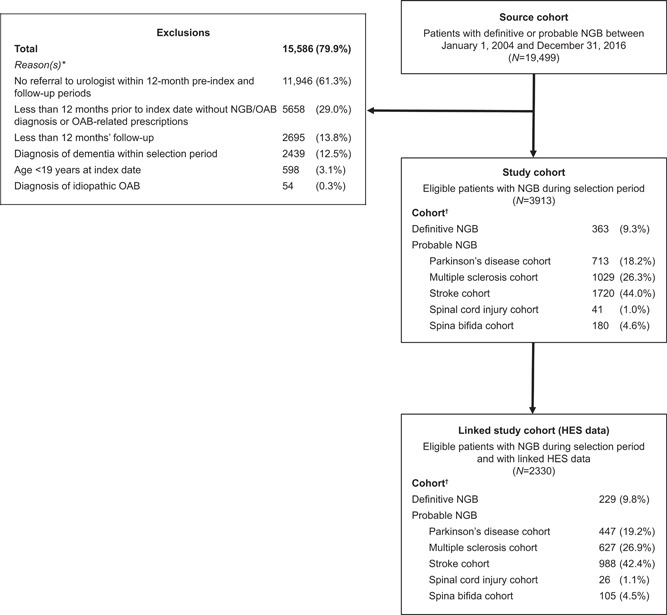
Patient selection flowchart. HES, Hospital Episodes Statistics; NGB, neurogenic bladder; OAB, overactive bladder. *Patients may have been excluded for more than one reason. ^†^Patients may have been eligible for more than one cohort

Between 1 January 2004 and 31 December 2016, 19 499 patients with definitive or probable NGB were identified (Figure 1). After applying the predefined eligibility criteria, 15 586 (79.9%) patients were excluded, most commonly because they were not referred to a urologist (n = 11 946, 61.3%) or because the preindex period was <12 months (n = 5658, 29.0%). The remaining 3913 (20.1%) patients constituted the study cohort, of whom 363 (9.3%) patients had definitive NGB and 3550 (90.7%) patients had probable NGB. Patients with probable NGB were stratified into the following cohorts based on their underlying neurological condition (note: groups were not mutually exclusive): stroke (n = 1720); multiple sclerosis (n = 1029); Parkinson's disease (n = 713); spina bifida (n = 180); and spinal cord injury (n = 41). Approximately 50% of the study cohort (n = 2330 patients) had data linked to the HES database.

The study cohort had a mean age of 61.7 (standard deviation [SD], 16.3) years, and 59.6% were men (Table 1). Comorbidities, assessed by the number of different drug classes prescribed, were common (mean 8.6 [SD, 7.6]), and occurred more frequently among older than younger patients (mean 10.0 [SD, 7.3] vs 7.4 [SD, 7.7]) (Table S3).

**Table 1:** Demographic, clinical characteristics, and drug utilization before or at index date (overall study cohort and by underlying neurological condition)

Variable			Probable NGB					All (N = 3913)
		Definitive NGB (N = 363)	PD cohort	MS cohort	STK cohort	SCI cohort	SB cohort	
			(N = 713)	(N = 1029)	(N = 1720)	(N = 41)	(N = 180)	
Age at index date, y	Mean (SD)	48.3 (15.9)	70.7 (9.2)	48.7 (11.8)	70.3 (11.6)	46.6 (14.8)	36.1 (11.9)	61.7 (16.3)
Sex, n (%)	Male	199 (54.8)	535 (75.0)	357 (34.7)	1188 (69.1)	31 (75.6)	90 (50.0)	2334 (59.6)
	Female	164 (45.2)	178 (25.0)	672 (65.3)	532 (30.9)	10 (24.4)	90 (50.0)	1579 (40.4)
Time from diagnosis of NGB/underlying conditions and OAB diagnosis/drug prescription, days	No. of valid values	135	711	1017	1715	37	135	3678
	Mean (SD)	846 (1064)	1034 (1008)	1096 (1010)	1029 (986)	458 (520)	4149 (4161)	1140 (1353)
Comorbidities within the 12‐mo pre‐index period, (using BNF headers/codes)	Mean (SD)	9.6 (9.1)	9.1 (6.9)	6.5 (7.1)	9.3 (7.6)	7.1 (8.6)	11.0 (8.9)	8.6 (7.6)
Polypharmacy at index date (using substances)	Mean (SD)	4.6 (5.0)	5.6 (4.2)	3.3 (3.7)	5.7 (4.6)	3.6 (4.8)	5.4 (4.8)	5.0 (4.4)
ACB score^a^	Mean (SD)	2.9 (4.5)	6.8 (5.9)	6.0 (4.9)	7.6 (6.3)	6.9 (7.1)	4.6 (4.7)	6.6 (5.9)
Prescriptions for anticholinergic drugs within the 12‐mo preindex period,^b^ n (%)	Any	185 (51.0)	408 (57.2)	465 (45.2)	982 (57.1)	17 (41.5)	144 (80.0)	2137 (54.6)
	1	68 (18.7)	172 (24.1)	223 (21.7)	384 (22.3)	6 (14.6)	63 (35.0)	893 (22.8)
	2	49 (13.5)	116 (16.3)	133 (12.9)	263 (15.3)	4 (9.8)	34 (18.9)	580 (14.8)
	3	22 (6.1)	47 (6.6)	59 (5.7)	154 (9.0)	3 (7.3)	22 (12.2)	298 (7.6)
	≥4	46 (12.7)	73 (10.2)	50 (4.9)	181 (10.5)	4 (9.8)	25 (13.9)	366 (9.4)
OAB drug at index date, *n* (%)	Yes	37 (10.2)	614 (86.1)	885 (86.0)	1515 (88.1)	34 (82.9)	131 (72.8)	3175 (81.1)
	Darifenacin	0	8 (1.1)	2 (0.2)	4 (0.2)	0	0	14 (0.4)
	Fesoterodine	0	20 (2.8)	31 (3.0)	49 (2.8)	2 (4.9)	2 (1.1)	104 (2.7)
	Flavoxate	0	12 (1.7)	11 (1.1)	17 (1.0)	0	3 (1.7)	43 (1.1)
	Mirabegron	0	19 (2.7)	9 (0.9)	19 (1.1)	0	1 (0.6)	48 (1.2)
	Oxybutynin ER	3 (0.8)	33 (4.6)	78 (7.6)	96 (5.6)	12 (29.3)	14 (7.8)	233 (6.0)
	Oxybutynin IR	12 (3.3)	119 (16.7)	265 (25.8)	380 (22.1)	6 (14.6)	31 (17.2)	803 (20.5)
	Propiverine	1 (0.3)	5 (0.7)	11 (1.1)	10 (0.6)	1 (2.4)	1 (0.6)	28 (0.7)
	Solifenacin	10 (2.8)	208 (29.2)	243 (23.6)	495 (28.8)	8 (19.5)	41 (22.8)	992 (25.4)
	Tolterodine	7 (1.9)	143 (20.1)	194 (18.9)	349 (20.3)	4 (9.8)	36 (20.0)	723 (18.5)
	Trospium	4 (1.1)	47 (6.6)	41 (4.0)	96 (5.6)	1 (2.4)	2 (1.1)	187 (4.8)

Abbreviations: ACB, anticholinergic cognitive burden; BNF, British National Formulary; ER, extended release; IR, immediate release; MS, multiple sclerosis; NGB, neurogenic bladder; OAB, overactive bladder; PD, Parkinson's disease; SB, spina bifida; SCI, spinal cord injury; SD, standard deviation; STK, stroke.

^a^ Prescribed medications with anticholinergic activity and negative cognitive effects were identified from Aging Brain Care^15^ and categorized according to the following scale: no anticholinergic activity (0) to definite/high anticholinergic activity (3).^14^ ACB score is the total score for all drugs prescribed to an individual patient. ACB score was calculated within 1 mo before and after the first OAB/NGB diagnosis or OAB drug prescription date. Note: over‐the‐counter medications were not included.

^b^ Identified from Aging Brain Care.^15^

### 3.2 Drug utilization

Two‐thousand one‐hundred and thirty‐seven (54.6%) patients received ≥1 prescription for drugs with anticholinergic activity before the index period (Table 1). The drugs with at least partial anticholinergic activity were not solely the OAB‐therapeutic antimuscarinics. Those most commonly prescribed were solifenacin (8.5%), warfarin (weakly anticholinergic, 7.9%), furosemide (weakly anticholinergic, 7.6%), amitriptyline (7.2%), oxybutynin (7.1%), codeine/paracetamol (weakly anticholinergic, 6.9%), and tolterodine (6.0%) Table S4). The mean ACB score, a measure of cumulative anticholinergic burden, was 6.6 (SD, 5.9) in the study cohort, and ranged from 2.9 (definite NGB cohort) to 7.6 (stroke cohort) (Table 1), but was similar between age and sex subgroups (Table S3).

At index date, 3175 (81.1%) patients were receiving an oral antimuscarinic agent or mirabegron. The most commonly prescribed agents were solifenacin (n = 992, 25.4%), oxybutynin immediate‐release (n = 803, 20.5%), tolterodine (n = 723, 18.5%), and oxybutynin extended‐release (n = 233, 6.0%) (Table 1). Other agents (including mirabegron) were each prescribed to <5% of patients.

### 3.3 During 12‐month follow‐up

Most of the study cohort (92.2%) received ≥ 1 prescription for an oral antimuscarinic agent or mirabegron over the 12‐month follow‐up period (Table 2); the mean number of prescriptions per individual was 6.9 (SD, 8.2). A notable exception was the definitive NGB cohort, where only 29.8% of patients received ≥ 1 prescription for these agents and had an average of 1.6 (SD, 3.5) prescriptions per individual. The mean cumulative number of days’ supply of OAB drugs was 203 (SD, 211) per individual in the study cohort. Overall, 312 (8.0%) patients were prescribed a combination of OAB drugs; the most common combinations included solifenacin, tolterodine, and oxybutynin (Table S5). Approximately half the cohort (53.9%) had prescriptions for antibiotics to treat UTIs, and 997 (25.5%) patients had prescriptions for α‐adrenergic antagonists or 5α‐reductase inhibitors (Table 2). Drug utilization patterns were similar between age and sex subgroups, except for α‐adrenergic antagonists/5α‐reductase inhibitors were prescribed more often in men (40.7% vs women, 3.0%) and older patients (37.4% vs younger, 14.5%) (Table S6).

**Table 2:** Drug utilization during the 12‐mo follow‐up period (overall study cohort and by underlying neurological condition)

Variable		Definitive NGB (N = 363)	Probable NGB					All (N = 3913)
			PD cohort	MS cohort	STK cohort	SCI cohort	SB cohort	
			(N = 713)	(N = 1029)	(N = 1720)	(N = 41)	(N = 180)	
Number of oral OAB drug prescriptions	Mean (SD)	1.6 (3.5)	7.6 (8.1)	7.1 (7.3)	7.5 (9.1)	9.0 (5.6)	5.0 (6.3)	6.9 (8.2)
Number of oral OAB drug prescriptions, n (%)	0	255 (70.2)	11 (1.5)	35 (3.4)	29 (1.7)	4 (9.8)	44 (24.4)	307 (7.8)
	1‐4	58 (16.0)	304 (42.6)	412 (40.0)	759 (44.1)	5 (12.2)	63 (35.0)	1571 (40.1)
	5‐9	31 (8.5)	160 (22.4)	275 (26.7)	416 (24.2)	13 (31.7)	32 (17.8)	911 (23.3)
	10‐14	14 (3.9)	177 (24.8)	243 (23.6)	370 (21.5)	13 (31.7)	37 (20.6)	841 (21.5)
	15‐44	5 (1.4)	51 (7.2)	52 (5.1)	117 (6.8)	6 (14.6)	3 (1.7)	231 (5.9)
	≥45	0	10 (1.4)	12 (1.2)	29 (1.7)	0	1 (0.6)	52 (1.3)
Cumulative number of days’ supply of oral OAB drugs	Mean (SD)	50.5 (112.5)	221.5 (202.3)	222.2 (188.5)	210.4 (232.6)	273.2 (158.5)	155.0 (155.6)	202.9 (210.9)
Cumulative number of days’ supply of oral OAB drugs, n (%)	0‐29	272 (74.9)	101 (14.2)	155 (15.1)	283 (16.5)	7 (17.1)	66 (36.7)	804 (20.5)
	30‐119	40 (11.0)	190 (26.6)	250 (24.3)	455 (26.5)	2 (4.9)	34 (18.9)	952 (24.3)
	120‐349	32 (8.8)	201 (28.2)	318 (30.9)	515 (29.9)	15 (36.6)	44 (24.4)	1107 (28.3)
	350‐549	18 (5.0)	211 (29.6)	292 (28.4)	445 (25.9)	17 (41.5)	36 (20.0)	1003 (25.6)
	≥550	1 (0.3)	10 (1.4)	14 (1.4)	22 (1.3)	0	0	47 (1.2)
Oral OAB drug combination use, n (%)	Yes	10 (2.8)	58 (8.1)	105 (10.2)	130 (7.6)	2 (4.9)	11 (6.1)	312 (8.0)
Number of prescriptions for antibiotics for UTI^a^	Mean (SD)	2.7 (4.3)	1.7 (3.3)	2.4 (4.2)	2.1 (3.8)	3.5 (4.9)	3.1 (6.2)	2.2 (4.0)
Number of prescriptions for antibiotics for UTI^a^, n (%)	0	159 (43.8)	371 (52.0)	473 (46.0)	768 (44.7)	20 (48.8)	71 (39.4)	1803 (46.1)
	1‐4	130 (35.8)	261 (36.6)	384 (37.3)	713 (41.5)	8 (19.5)	70 (38.9)	1520 (38.8)
	5‐9	42 (11.6)	55 (7.7)	97 (9.4)	144 (8.4)	9 (22.0)	25 (13.9)	352 (9.0)
	10‐14	23 (6.3)	22 (3.1)	49 (4.8)	54 (3.1)	2 (4.9)	9 (5.0)	155 (4.0)
	15‐19	9 (2.5)	4 (0.6)	26 (2.5)	40 (2.3)	2 (4.9)	4 (2.2)	81 (2.1)
	≥20	0	0	0	1 (0.1)	0	1 (0.6)	2 (0.1)
Number of α‐adrenergic antagonists or 5‐ARI prescriptions^b^	Mean (SD)	0.8 (2.9)	4.3 (9.8)	0.6 (2.4)	4.3 (9.5)	0.6 (3.8)	0.6 (2.7)	2.9 (8.0)
Number of α‐adrenergic antagonists or 5‐ARI prescriptions,^b^ n (%)	0	321 (88.4)	448 (62.8)	958 (93.1)	1107 (64.4)	39 (95.1)	165 (91.7)	2916 (74.5)
	1‐4	14 (3.9)	63 (8.8)	18 (1.7)	126 (7.3)	1 (2.4)	6 (3.3)	223 (5.7)
	5‐9	16 (4.4)	76 (10.7)	26 (2.5)	177 (10.3)	0	4 (2.2)	296 (7.6)
	10‐14	10 (2.8)	76 (10.7)	23 (2.2)	190 (11.0)	0	4 (2.2)	300 (7.7)
	15‐19	2 (0.6)	41 (5.8)	4 (0.4)	102 (5.9)	1 (2.4)	1 (0.6)	151 (3.9)
	≥20	0	9 (1.3)	0	18 (1.0)	0	0	27 (0.7)

Abbreviations: 5‐ARI, 5α‐reductase inhibitors; MS, multiple sclerosis; NGB, neurogenic bladder; OAB, overactive bladder; PD, Parkinson's disease; SB, spina bifida; SCI, spinal cord injury; SD, standard deviation; STK, stroke; UTI, urinary tract infection.

^a^ Trimethoprim, ciprofloxacin, nitrofurantoin, amoxicillin, amoxicillin/clavulanic acid at any dosage.

^b^ Doxazosin, tamsulosin, alfuzosin, terazosin, finasteride, dutasteride at any dosage.

### 3.4 Complications

During the 12‐month follow‐up period, 558 (14.3%) patients had UTIs and 557 (14.2%) patients experienced urinary incontinence (Table 3). Other complications were each recorded in <3% of patients. UTIs and urinary incontinence were the most commonly reported complications regardless of underlying neurological condition (Table 3), age, or sex (Table S7).

**Table 3:** Complications during the 12‐mo follow‐up period (overall study cohort and by underlying neurological condition)

Complication,^a^ n (%)	Definitive NGB (N = 363)	Probable NGB					All (N = 3913)
		PD cohort (N = 713)	MS cohort (N = 1029)	STK cohort (N = 1720)	SCI cohort (N = 41)	SB cohort (N = 180)	
Urinary tract infection	71 (19.6)	72 (10.1)	153 (14.9)	237 (13.8)	14 (34.1)	35 (19.4)	558 (14.3)
Urinary incontinence	31 (8.5)	119 (16.7)	141 (13.7)	260 (15.1)	2 (4.9)	21 (11.7)	557 (14.2)
Urinary retention	13 (3.6)	21 (2.9)	19 (1.8)	45 (2.6)	0	1 (0.6)	96 (2.5)
Sepsis/septicemia	4 (1.1)	5 (0.7)	7 (0.7)	18 (1.0)	1 (2.4)	2 (1.1)	34 (0.9)
Renal failure (acute or other)	13 (3.6)	5 (0.7)	2 (0.2)	7 (0.4)	0	5 (2.8)	27 (0.7)
Hydronephrosis	6 (1.7)	3 (0.4)	1 (0.1)	3 (0.2)	0	3 (1.7)	14 (0.4)
Obstructive uropathy	0	0	0	0	0	1 (0.6)	1 (0.0)

Abbreviations: MS, multiple sclerosis; NGB, neurogenic bladder; PD, Parkinson's disease; SB, spina bifida; SCI, spinal cord injury; STK, stroke.

^a^ Each complication was identified from medical records using prespecified read codes.

### 3.5 Healthcare resource use and costs

Healthcare resource use and costs during the 12‐month follow‐up period are presented in Table 4, and by age and sex in Table S8. All patients had ≥1 GP consultation for any cause, with a mean of 67.7 (SD, 42.6) visits per individual over the 12‐month follow‐up period. Patients with stroke or spinal cord injury visited their GP more often (76.3 and 75.5, respectively) than other cohorts (range, 49.9‐69.7). The estimated overall mean cost of GP consultations was £1448 (SD, £967) per individual. Overall, 2.5% of the study cohort had ≥1 urodynamic test (mean cost, £179 [SD, 94] per individual), 8.8% underwent cystoscopy (£171 [SD, 66] per individual), and 2.1% had urology‐related imaging (£101 [SD, 83] per individual). Only 14 (0.4%) patients in the study cohort were prescribed incontinence pads (mean cost, £40 [SD, 47] per individual). Almost half of the study cohort (46.7%) visited a specialist (urologist or gynecologist) over the 12‐month follow‐up period, with a mean 2.3 (SD, 1.7) visits at a mean cost of £253 (SD, 186) per individual; most visits (90.8%) were to a urologist rather than a gynecologist (Table 4). At least one procedure or surgical intervention was performed in 5.7% of the study cohort at a mean cost of £2285 (SD, £3919) per individual. Overall, 11.0% (n = 431) of the study cohort were hospitalized for a mean of 12.5 days during the 12‐month follow‐up period; 17.4% (75 of 431) of hospitalized patients were admitted after renal failure. Hospital admissions were more common in the definite NGB and spinal cord injury cohorts (20.1% and 19.5%, respectively) compared to other cohorts (range, 6.7 to 12.0%), but were similar between age and sex subgroups. The mean costs for hospitalization were £6256 (SD, £13,473) per individual.

**Table 4:** Resource use and costs during the 12‐mo follow‐up period (overall study cohort and by underlying neurological condition)

Resource	Variable		Definitive NGB(N = 363)	Probable NGB						
				PD cohort	MS cohort	STK cohort	SCI cohort	SB cohort		All (N = 3913)
				(N = 713)	(N = 1029)	(N = 1720)	(N = 41)	(N = 180)		
GP consultations (all‐cause)^a^	≥1 visit	*n* (%)	363 (100)	713 (100)	1029 (100)	1720 (100)	41 (100)	180 (100)		3913 (100)
	Visits, n	Mean (SD)	55.2 (43.8)	69.7 (39.4)	57.4 (39.4)	76.3 (44.0)	75.5 (54.1)	49.9 (35.1)		67.7 (42.6)
	Cost, £	Mean (SD)	1181 (985)	1501 (911)	1218 (880)	1627 (1005)	1523 (1158)	1104 (809)		1448 (967)
		Median (range)	937 (46‐6530)	1333 (114‐8099)	1025 (37‐9382)	1398 (46‐10776)	1423 (46‐6512)	963 (37‐3925)		1244 (37‐10776)
Specialist visits (urologist/gynecologist)	≥1 visit (overall)	*n* (%)	184 (50.7)	342 (48.0)	396 (38.5)	859 (49.9)	23 (56.1)	92 (51.1)		1828 (46.7)
	Urologist		174 (47.9)	328 (46.0)	367 (35.7)	820 (47.7)	20 (48.8)	85 (47.2)		1729 (44.2)
	Gynecologist		18 (5.0)	20 (2.8)	50 (4.9)	79 (4.6)	5 (12.2)	9 (5.0)		175 (4.5)
	Visits, n (overall)	Mean (SD)	2.4 (1.6)	2.1 (1.4)	2.1 (1.4)	2.4 (1.8)	2.1 (1.2)	2.0 (1.4)		2.3 (1.7)
	Urologist		2.3 (1.7)	2.0 (1.5)	1.9 (1.4)	2.2 (1.8)	1.9 (1.4)	1.7 (1.3)		2.1 (1.6)
	Gynecologist		0.2 (0.5)	0.1 (0.5)	0.2 (0.7)	0.2 (0.8)	0.2 (0.4)	0.3 (1.0)		0.2 (0.7)
	Cost, £ (overall)	Mean (SD)	270 (182)	235 (159)	236 (160)	267 (207)	235 (132)	225 (171)		253 (186)
		Median (range)	219 (109‐1094)	219 (109‐1313)	219 (109‐1079)	219 (109‐1641)	219 (109‐547)	141 (109‐955)		219 (109‐1641)
Incontinence pads	≥1 pad	*n* (%)	2 (0.6)	2 (0.3)	5 (0.5)	5 (0.3)	1 (2.4)	0		14 (0.4)
	Prescriptions, n	Mean (SD)	1.5 (0.7)	1.0 (0.0)	10.0 (7.0)	3.8 (4.2)	1.0 (⋯)	⋯		5.2 (6.0)
	Cost, £	Mean (SD)	13 (4)	8 (3)	88 (49)	10 (8)	50 (⋯)	⋯		40 (47)
		Median (range)	13 (10‐16)	8 (6‐10)	120 (16‐126)	6 (4‐24)	50 (50‐50)	⋯		14 (4‐126)
Urodynamics	≥1 test	*n* (%)	9 (2.5)	11 (1.5)	27 (2.6)	51 (3.0)	0	3 (1.7)		98 (2.5)
	Tests, n	Mean (SD)	1.1 (0.3)	1.8 (0.6)	1.7 (1.1)	1.3 (0.5)	⋯	1.0 (0.0)		1.4 (0.8)
	Cost, £	Mean (SD)	140 (42)	229 (76)	215 (135)	158 (66)	⋯	126 (0)		179 (94)
		Median (range)	126 (126‐252)	252 (126‐378)	126 (126‐630)	126 (126‐378)	⋯	126 (126‐126)		126 (126‐630)
Cytoscopy	≥1 test	*n* (%)	55 (15.2)	74 (10.4)	58 (5.6)	147 (8.5)	4 (9.8)	22 (12.2)		343 (8.8)
	Tests, n	Mean (SD)	1.2 (0.6)	1.1 (0.3)	1.1 (0.3)	1.2 (0.5)	1.0 (0.0)	1.2 (0.5)		1.2 (0.5)
	Cost, £	Mean (SD)	178 (87)	166 (50)	156 (37)	180 (78)	146 (0)	173 (73)		171 (66)
		Median (range)	146 (146‐584)	146 (146‐292)	146 (146‐292)	146 (146‐584)	146 (146‐146)	146 (146‐438)		146 (146‐584)
Imaging^b^	≥1 test	*n* (%)	9 (2.5)	9 (1.3)	24 (2.3)	37 (2.2)	2 (4.9)	5 (2.8)		83 (2.1)
	Tests, n	Mean (SD)	1.0 (0.0)	1.0 (0.0)	1.1 (0.3)	1.1 (0.3)	1.0 (0.0)	1.0 (0.0)		1.1 (0.3)
	Cost, £	Mean (SD)	73 (72)	64 (76)	138 (79)	87 (85)	144 (0)	92 (85)		101 (83)
		Median (range)	83 (0‐144)	0 (0‐144)	144 (0‐288)	144 (0‐288)	144 (144‐144)	144 (0‐173)		144 (0‐288)
Procedures and surgical interventions (urology)^c^ ^,^ ^d^	≥1 procedure	*n* (%)	44 (12.1)	30 (4.2)	52 (5.1)	104 (6.0)	7 (17.1)	2 (1.1)		223 (5.7)
	Procedures, n	Mean (SD)	1.4 (0.8)	1.6 (0.9)	1.4 (0.6)	1.3 (0.6)	1.3 (0.5)	1.0 (0.0)		1.4 (0.7)
	Cost, £	Mean (SD)	3408 (7295)	2035 (1774)	1999 (2564)	2472 (5142)	789 (505)	2752 (3143)		2285 (3919)
		Median (range)	1513 (228‐47419)	1529 (228‐6802)	1163 (168‐14934)	896 (220‐47419)	693 (168‐1410)	2752 (530‐4975)		1123 (168‐47419)
Hospitalizations (urology)^d^ ^,^ ^e^	≥1 hospitalization	*n* (%)	73 (20.1)	78 (10.9)	84 (8.2)	206 (12.0)	8 (19.5)	12 (6.7)		431 (11.0)
	Hospitalizations, n	Mean (SD)	2.1 (2.0)	1.4 (0.9)	1.7 (1.5)	1.5 (0.9)	1.6 (1.4)	2.3 (2.2)		1.6 (1.3)
	Admitted days, n	Mean (SD)	13.7 (38.4)	9.5 (14.5)	15.5 (32.8)	12.7 (23.5)	7.1 (9.6)	3.3 (6.1)		12.5 (26.6)
	Cost, £	Mean (SD)	8052 (20759)	5885 (8486)	6226 (14321)	5914 (11056)	2449 (2630)	7217 (15369)		6256 (13473)
		Median (range)	1943 (265‐163720)	2604 (220‐50521)	1794 (162‐83317)	3146 (162‐134885)	1648 (220‐8335)	2134 (265‐55306)		2590 (162‐163720)
Total	Cost, £	Mean (SD)	3379 (10676)	2356 (3850)	1923 (5034)	2624 (4944)	2290 (2025)	1757 (4347)		2395 (5413)
		Median (range)	1308 (46‐166644)	1546 (160‐59696)	1180 (37‐94393)	1667 (46‐137794)	1858 (120‐10171)	1182 (37‐57354)		1458 (37‐166644)

Abbreviations: GP, general practitioner; MS, multiple sclerosis; NGB, neurogenic bladder; PD, Parkinson's disease; SB, spina bifida; SCI, spinal cord injury; SD, standard deviation; STK, stroke.

^a^ Includes surgery and clinical consultations, home visits, out‐of‐hours visits, and telephone consultations.

^b^ Cystography, ultrasound, computed tomography, x‐ray, magnetic resonance imaging, and other diagnostic imaging of bladder, spine, genitourinary system, pelvis or abdomen.

^c^ Includes intermittent catheterization, indwelling catheterization, botulinum toxin A injections, sacral nerve stimulation, bladder augmentation, sling procedures, and artificial urinary sphincter.

^d^ Derived from the Hospital Episode Statistics (HES) database. Patients without linked data available from HES were assumed to have not utilized these resources. Numbers of hospital admissions or procedures and surgical interventions were calculated among patients who had ≥1 resource use item.

^e^ Admissions related to a urological disease, or to PD, MS, SCI, STK, SB with a related urological disease. Urological diseases included pyelonephritis, sepsis, hydronephrosis, uropathy, renal failure, chronic kidney disease, calculus, cystitis, neuropathic bladder, urethritis, urinary tract infection, proteinuria, incontinence, and retention.

### 3.6 Sensitivity analysis

The primary objectives of the study were reanalyzed using an alternative definition for patients with probable NGB (ie, diagnoses for the underlying neurological condition or OAB diagnosis/OAB drug prescription could be in any order). The results were similar to those of the base‐case analysis (data not shown).

## 4 DISCUSSION

This study provides detailed descriptive information about patients with NGB and, to our knowledge, is the first study to characterize this patient population in the UK. Over the 13‐year study selection period, we identified 363 patients with a definitive diagnosis of NGB and a further 3550 patients with probable NGB based on proxy inclusion criteria. The high number of probable cases suggests that many patients with NGB may not be formally diagnosed in the UK, and is possibly indicative of low awareness of the condition among GPs and neurologists, or limitations in data coding practices. A notable feature of patient selection in our study was the requirement for at least one referral to a urologist, a criterion intended to reduce the risk of including non‐NGB patients. This requirement led to the exclusion of many patients (61%) from the original source cohort, suggesting that our final study cohort may be an underestimate of the true size of the NGB population. It also highlights that primary care providers may take responsibility for much of the care of patients with NGB. We recognize also that the requirement for a urologist referral may have removed some patients with a lower burden of illness from our study, and suggest that this should be considered when interpreting the data.

Our findings suggest that patients with NGB have multiple comorbidities and complications. Urinary incontinence and UTIs were common, and more than half (54%) of our study population received prescriptions for UTI‐specific antibiotics. Serious complications (renal failure and sepsis) were rare and affected less than 1% of patients each, although observation of these events is likely limited by the short 12‐month follow‐up period. A similar spectrum of complications was documented in a large US claims database study of patients with NGB (n = 46 271),^1^ albeit at much higher frequencies than in our study. For example, UTIs, urinary retention, and sepsis/septicemia were reported in 33%, 14%, and 4% of patients in the US study,^1^ respectively, compared with 14%, 2%, and 1% in our study. As key design features, including duration of follow‐up, were similar between studies, the reason for the marked disparity between reporting rates is unknown.

The drug utilization patterns documented in our study were consistent with current NGB treatment guidelines.^4^, ^8^ Antimuscarinic agents are recommended as first‐line pharmacological therapy for NDO,^4^, ^8^ and most (92%) patients in our study had ≥1 prescription for an antimuscarinic agent or mirabegron over the 12‐month study period. It is possible that this rate may be elevated by the requirement for an OAB prescription for inclusion into the probable NGB cohort; the prescribing rate was lower (30%) in the definite NGB cohort whose selection was based on NGB diagnosis alone. It is also notable that some of this prescribing were off‐label as several of these agents do not have marketing authorization for the treatment for NGB in the UK. Antimuscarinic combinations, also supported by treatment guidelines,^8^ were prescribed in 8% of patients. Mirabegron was prescribed infrequently (about 1% of patients), although its use in NGB is not currently supported by treatment guidelines^4^, ^8^ and it was introduced in the UK only towards the end of the study selection period. One‐quarter of the study cohort also received prescriptions for α‐adrenergic antagonists or 5α‐reductase inhibitors; α‐adrenergic antagonists are recommended for bladder outlet resistance in NGB^4^, ^8^ and may have contributed to some of this prescribing.

Neurological patients may be particularly susceptible to the unwanted central actions of some drugs because the integrity of the blood‐brain barrier can be disrupted by the disease process.^2^, ^9^ The National Institute for Health and Care Excellence (NICE) suggests that the potential for CNS‐related side effects with agents known to cross the blood‐brain barrier (eg, oxybutynin) should be considered when prescribing an antimuscarinic agent for NGB^10^ yet, in our study, oxybutynin was prescribed to approximately 25% of patients. Furthermore, other drugs with anticholinergic activity and the potential for causing negative cognitive effects were commonly prescribed, and the cumulative ACB in our study population at baseline was high (mean ACB score 6.6, where a score ≥3 is considered to be clinically relevant^14^). The implications of overprescribing drugs with anticholinergic effects are well documented; each 1‐point increase on the ACB scale is associated with a 13% increase in the risk of cognitive impairment, and an 11% increase in the likelihood of inpatient admission.^19^ Increased prescriber awareness of drugs with anticholinergic effects may help to reduce the risk of unwanted CNS events in these patients.

High levels of healthcare resource use were evident in our study population. On average, patients visited or made contact with their general practice 68 times over a 12‐month period. Patients also visited a specialist twice on average and had one urology‐related hospital admission and one surgical procedure over a 12‐month period. An unexpected finding was the very low rate of prescribing for incontinence pads (0.4%), which was at odds with the reporting rate for urinary incontinence (14%) in our study. It seems likely that patients purchased pads as an out‐of‐pocket expense rather than obtaining them by prescription. After applying unit costs from standard UK sources, the average costs for healthcare resource utilization (excluding drugs) across the overall study cohort was an estimated £2395 per individual over 12 months. To our knowledge, this is first study to provide a comprehensive evaluation of healthcare‐related costs in patients with NGB, as previous cost‐of‐illness studies have focused on emergency department admissions alone.^20^, ^21^

The patient selection criteria adopted in our study (ie, proxy criteria to identify probable cases of NGB [based on diagnosis of a neurological condition plus a diagnosis of OAB/prescription of OAB drug] and requirement for a urologist referral) may introduce selection bias and limit the generalizability of our findings. However, when studying a condition such as NBG with proposed underdiagnoses and low awareness in some clinician groups, the selection process must adopt an appropriate level of specificity and sensitivity. Further, the drug choices reported are likely to have been influenced by NICE and may differ from other countries. The main limitations of our study were its retrospective design and the absence of a control cohort to compare outcomes against. Cost estimates are likely to be conservative as the unit costs and NHS tariffs used in our study did not capture all relevant direct medical costs (eg, theater time), and drug acquisition costs were also not considered.

We suggest that future studies should include a longer follow‐up period and include less common neurological conditions associated with NGB (eg, cerebral palsy). Physician surveys, to better understand the rationale for prescribing decisions, as well as studies of cognitive function in patients receiving antimuscarinic agents would also be of interest.

## 5 CONCLUSIONS

Our findings suggest that NGB may be underrecognized among primary care providers in the UK. The burden of illness, healthcare needs and associated costs evident in our study population were considerable. Drug prescribing patterns were consistent with the symptoms and complications of NGB, although increased awareness and reduced prescribing of agents with anticholinergic effects may help to improve health outcomes in this vulnerable patient population.

## DISCLOSURE OF INTERESTS

AJ and MA worked full time at Astellas Pharma under a Knowledge Transfer Partnership (KTP) scheme with Manchester Metropolitan University (MMU) when the research was conducted; JN was an employee of Astellas Pharma when the research was conducted; FF reports receiving grants from Astellas during the conduct of the study (project was funded as part of MMU/Astellas KTP), and grants from Astellas outside the submitted work; ES and NC are employees of Astellas Pharma; RA reports receiving grants from Astellas during the conduct of the study, and grants from Astellas outside the submitted work; DdR reports receiving grants from Astellas, Ferring and nonfinancial support from Pierre‐Fabre outside the submitted work; MJD reports nonfinancial support from Astellas during the conduct of the study, personal fees from Allergan, Astellas, and grants and personal fees from Ferring outside the submitted work.

## DATA AVAILABILITY

Access to anonymized individual participant level data will not be provided for this trial as it meets one or more of the exceptions described on http://www.clinicalstudydatarequest.com under "Sponsor Specific Details for Astellas."

## Supporting information

Supporting information
