# Supplementary material for: Drug utilization patterns and healthcare resource use and costs in patients with neurogenic bladder in the United Kingdom: A retrospective primary care database study
Source: Neurourol Urodyn. 2019 Mar 28;38(5):1278–89. doi: 10.1002/nau.23981 (PMC6850049; doi:10.1002/nau.23981)
Supplement: Supplementary file 1 — Supporting information [file NAU-38-1278-s001.docx]

**Supporting Information**

**Table of contents**

**Appendix**. Anticholinergic Cognitive Burden Scale 2

**Table S1.** Study endpoints and definitions 3

**Table S2.** Unit costs and cost calculations 5

**Table S3.** Demographic and clinical characteristics by age and gender 6

**Table S4.** Anticholinergic drug utilisation during the 12-month follow-up period ….….…7

**Table S5.** OAB drug combinations during the 12-month follow-up period (overall study cohort) 9

**Table S6.** Drug utilisation during the 12-month follow-up period by age and gender 10

**Table S7.** Complications during the 12-month follow-up period by age and gender 11

**Table S8.** Resource use and costs during the 12-month follow-up period by age and gender 12

**Figure S1.** Patient selection criteria 14

**References** 15

**Appendix**

**Anticholinergic Cognitive Burden Scale**

The Anticholinergic Cognitive Burden (ACB) Scale was developed by Boustani et al.^1^ as a practical tool to capture the cumulative ACB for an individual patient as a result of their medications. Studies of drugs with anticholinergic activity that showed negative cognitive effects, including delirium, mild cognitive impairment, dementia or cognitive decline, were identified from a literature search. An expert interdisciplinary team then categorised the drugs and assigned an ACB score according to their negative cognitive anticholinergic effects (see Table below for details).^1,2^ The total added score of different drugs taken by the patient determines their accumulative ACB. A total ACB Scale score of ≥3 is considered clinically relevant. For the present study, the list from Aging Brain Care 2012 was used to identify drugs with anticholinergic effects and to assign the ACB score for each drug.^2^

| **Category** | **Criteria** | **ACB score** |
| --- | --- | --- |
| No anticholinergic effects |  | 0 |
| Possible anticholinergic effects | Evidence of *in vitro* anticholinergic activity or affinity for muscarinic receptors but with no clinically relevant negative cognitive effects | 1 |
| Definite anticholinergic effects | Evidence from literature, prescriber’s information, or expert opinion of clinical anticholinergic effect | 2 |
| Definite anticholinergic effects | Evidence from literature, expert opinion, or prescribers information that medication may cause delirium | 3 |

TABLE S1 Study endpoints and definitions

| **Endpoint** | **Variable** | **Definition** |
| --- | --- | --- |
| ***Primary endpoints*** |  |  |
| Patient demographics | Age at index date  Sex |  |
| Comorbidities | Number of distinct BNF headers within the 12-month pre‑index period |  |
| Diagnosis of NGB or underlying neurological condition | Diagnosis of NGB or OAB preceded by diagnosis of underlying neurological condition (PD, MS, SCI, STK or SB) |  |
|  | Duration between diagnosis of NGB/underlying condition and OAB diagnosis/OAB drug prescription | Calculated as the difference between the date of OAB diagnosis/OAB drug prescription (whichever comes first), and the date of NGB/underlying condition (whichever comes first) |
| Complications | Complications over 12-month follow-up period, ie, UTI, incontinence, sepsis/septicaemia, urinary retention, obstructive uropathy, renal failure (acute and other), hydronephrosis |  |
| Drug utilisation at index date | ACB score | Sum of ACB scores for all anticholinergic medicines prescribed at time of the first OAB/NGB diagnosis or OAB drug prescription |
|  | Polypharmacy | Defined by the number of distinct BNF headers within 30 days before and after index date, and the number of distinct substance names within 30 days before and after index date |
| Drug utilisation during the 12‑month follow-up period | OAB drug prescribed |  |
|  | Number of OAB prescriptions |  |
|  | Cumulative number of days’ supply of OAB drugs | Sum of days’ supply of all prescriptions occurring over the 12‑month post-index period. If a prescription date fell within the 12-month post-index period but ended outside it, only days’ supply within the follow-up period were considered |
|  | Total quantity of OAB drug (mg) | Sum of all doses prescribed over the 12-month post-index period |
|  | Number of patients with concomitant use of two or more OAB drugs (combination use) | Patients were considered to having a combination of OAB drugs if at least two drugs overlapped for more than 30 days |
|  | Number of patients with intermittent catheter and indwelling catheter accompanying OAB drug use |  |
|  | Number of patients with prescriptions for α-adrenergic antagonist or 5-ARIs |  |
|  | Number of patients with prescriptions for antibiotics for UTI |  |
| ***Secondary endpoints*** |  |  |
| Healthcare resource use during the 12-month follow up period | Occurrence (yes/no) and number of outpatient referrals (CPRD) |  |
|  | Occurrence (yes/no) and number of outpatient referrals (urologists and gynaecologists) (CPRD) |  |
|  | Number of all-cause GP consultations (CPRD) |  |
|  | Number of pads used (CPRD) |  |
|  | Urological investigations/tests | Included: urinalysis; culture and assessment of post-void residual urine; cystoscopy; urodynamics; imaging (upper tract, spine) |
|  | Radiology (CPRD) | Radiology procedures used in NGB, ie, mercaptuacetyltriglycine (MAG, renal scan), ultrasound, computed tomography scan |
|  | Number of hospital admissions (urology) (HES) | All admissions associated with: a primary ICD-10 code related to a urological disease; a primary ICD-10 code related to PD, MS, SCI, STK, SB and a secondary ICD-10 code related to a urological disease |
|  | Number of procedures and operations performed (HES) | All events from the HES procedure dataset associated with an OPCS-4 code related to the following surgical interventions: intermittent catheterisation; indwelling catheterisation; injections of botulinum toxin A; sacral nerve stimulation; bladder augmentation; sling procedures; artificial urinary sphincter |
| Healthcare resource use costs during the 12-month follow-up period | Costs of primary care visits  Costs of secondary care referrals  Costs of incontinence pad use  Costs of diagnostic tests  Costs of laboratory tests, ie, costs of radiology  Costs of hospitalisations  Costs of procedures and operations performed | Healthcare resource use costs potentially related to NGB were estimated at the patient level by applying to each resource utilisation unit observed during the 12-month follow-up period the associated unit cost from the NHS perspective, and then summing costs by healthcare resource category |

5-ARI, 5α-reductase inhibitor; ACB, anticholinergic cognitive burden; BNF, British National Formulary; CPRD, Clinical Practice Research Datalink; GP, general practitioner; HES, Hospital Episode Statistics; ICD-10, International Classification of Diseases, 10th Revision; MS, multiple sclerosis; NGB, neurogenic bladder; OAB, overactive bladder; PD, Parkinson’s disease; SB, spina bifida; SCI, spinal cord injury; SD, standard deviation; STK, stroke; UTI, urinary tract infection.

TABLE S2 Unit costs and cost calculations

| **Variable** | **Unit costs** | **Source** |
| --- | --- | --- |
| ***Primary care (general practitioner)*** |  |  |
| Surgery consultation (lasting 9.22 minutes) | £37.00 | Curtis et al. 2017^3^ |
| Clinic | £68.80^a^ | Curtis et al. 2017^3^; Curtis 2014^4^ |
| Home visit | £45.60^b^ | Curtis et al. 2017^3^; Curtis 2014^4^ |
| Telephone consultation | £28.40^c^ | Curtis et al. 2017^3^; Curtis 2014^4^ |
| ***Specialists (outpatient attendance)*** |  |  |
| Urologist | £109.40 | NHS 2016–17^5^ |
| Gynaecologist | £140.93 | NHS 2016–17^5^ |
| ***Urological tests*** |  |  |
| Urodynamic test | £126.00 | NHS 2016–17^5^ |
| Cystoscopy | £146.00 | NHS 2016–17^5^ |
| ***Incontinence pads*** |  |  |
| One pad | £0.20^d^ | Age UK^6^ |
| ***Radiology*** |  |  |
| Ultrasound | £144.00 | NHS 2016–17^5^ |
| Computed tomography | £83.00 | NHS 2016–17^5^ |
| ***Procedures and surgical interventions*** | –^e^ | Adapted from NHS 2016–17^5^ |
| ***Hospital visits*** | –^e^ | Adapted from NHS 2016–17^5^ |

^a^17.2 minutes @ £4 per minute.

^b^11.4 minutes @ £4 per minute.

^c^7.1 minutes @ £4 per minute.

^d^Lille Healthcare Classic Pad PE Backed Maxi 1250 ml pack of 30 (£5.99 for 30 pads).

^e^For each episode, costs were derived from Healthcare Resource Group (HRG) tariffs published by the National Health Service (NHS) (2016/17) as follows: short episodes (LOS ≤ trimpoint), cost = T; long episodes (LOS > trimpoint), cost = T + (LOS - D) * E.

Where: T = combined day case/ordinary elective spell tariff; D = ordinary elective long-stay trimpoint (days); E = per day long-stay payment (for days exceeding trimpoint) (£); LOS = length of stay.

Missing HRG tariffs were collected from the national schedule of reference costs OR if not available, determined from previous years if available and an inflation rate was applied OR, if not available, imputed as £0.1.

TABLE S3 Demographic and clinical characteristics by age and gender

| **Variable** | | **19–65 years**  **(*N*=2038)** | **>65 years**  **(*N*=1875)** | **Male**  **(*N*=2334)** | **Female**  **(*N*=1579)** |
| --- | --- | --- | --- | --- | --- |
| Age at index date, years | Mean (SD) | 49 (12) | 76 (6) | 65 (15) | 57 (17) |
| Time from diagnosis of NGB/underlying conditions and OAB diagnosis/drug prescription, days | No. of valid values | 1854 | 1824 | 2192 | 1486 |
|  | Mean (SD) | 1348 (1679) | 929 (859) | 1135 (1313) | 1148 (1410) |
| Comorbidities within the 12‑month pre-index period, *n*  (using BNF headers/codes) | Mean (SD) | 7.4 (7.7) | 10.0 (7.3) | 8.3 (7.2) | 9.1 (8.3) |
| Comorbidities within the 12 month pre-index period, *n* (%)  (using BNF headers/codes) | 0 | 411 (20.2) | 209 (11.1) | 357 (15.3) | 263 (16.7) |
|  | 1–3 | 398 (19.5) | 135 (7.2) | 321 (13.8) | 212 (13.4) |
|  | 4–7 | 444 (21.8) | 437 (23.3) | 555 (23.8) | 326 (20.6) |
|  | 8–19 | 620 (30.4) | 891 (47.5) | 917 (39.3) | 594 (37.6) |
|  | ≥20 | 165 (8.1) | 203 (10.8) | 184 (7.9) | 184 (11.7) |
| Polypharmacy at index date, *n*  (using BNF substances) | Mean (SD) | 4.1 (4.4) | 6.0 (4.3) | 5.1 (4.3) | 4.8 (4.6) |
| Polypharmacy at index date, *n* (%)  (using BNF substances) | 0 | 510 (25.0) | 263 (14.0) | 448 (19.2) | 325 (20.6) |
|  | 1–3 | 620 (30.4) | 315 (16.8) | 518 (22.2) | 417 (26.4) |
|  | 4– 7 | 537 (26.3) | 675 (36.0) | 763 (32.7) | 449 (28.4) |
|  | 8–19 | 354 (17.4) | 614 (32.7) | 595 (25.5) | 373 (23.6) |
|  | ≥20 | 17 (0.8) | 8 (0.4) | 10 (0.4) | 15 (0.9) |
| ACB score^a^ | Mean (SD) | 6.3 (5.9) | 6.9 (5.7) | 6.5 (5.7) | 6.7 (6.1) |
| Prescriptions for anticholinergic drugs^b^ within the 12‑month pre-index period, *n* (%) | 0 | 1022 (50.1) | 1115 (59.5) | 1234 (52.9) | 903 (57.2) |
|  | 1 | 463 (22.7) | 430 (22.9) | 554 (23.7) | 339 (21.5) |
|  | 2 | 250 (12.3) | 330 (17.6) | 329 (14.1) | 251 (15.9) |
|  | 3 | 135 (6.6) | 163 (8.7) | 164 (7.0) | 134 (8.5) |
|  | ≥4 | 174 (8.5) | 192 (10.2) | 187 (8.0) | 179 (11.3) |

ACB, anticholinergic cognitive burden; BNF, British National Formulary; NGB, neurogenic bladder; OAB, overactive bladder; SD, standard deviation.

^a^Prescribed medications with anticholinergic activity and negative cognitive effects were identified from Aging Brain Care 2012^2^ and categorised according to the following scale: no anticholinergic activity (0) to definite/high anticholinergic activity (3).^1^ ACB score is the total score for all drugs prescribed to an individual patient. Note: over-the-counter medications were not included.

^b^Identified from Aging Brain Care 2012.

TABLE S4 Anticholinergic drug utilisation during the 12-month follow-up period

| **Anticholinergic drug** | **n (%)** | **Anticholinergic drug** | **n (%)** | **Anticholinergic drug** | **n (%)** |
| --- | --- | --- | --- | --- | --- |
| ***Solifenacin*** | 9228 (8.5) | Alverine | 359 (0.3) | Haloperidol | 59 (0.1) |
| Warfarin | 8550 (7.9) | ***Flavoxate*** | 353 (0.3) | Methocarbamol | 49 (0.1) |
| Furosemide | 8274 (7.6) | Propiverine | 321 (0.3) | Oxcarbazepine | 48 (0.0) |
| ***Amitriptyline*** | 7821 (7.2) | Hyoscine | 277 (0.3) | Alimemazine tartrate | 47 (0.0) |
| ***Oxybutynin*** | 7690 (7.1) | ***Olanzapine*** | 273 (0.3) | Buclizine / paracetamol / codeine | 47 (0.0) |
| Codeine / paracetamol | 7530 (6.9) | ***Nortriptyline*** | 270 (0.3) | Dicycloverine | 44 (0.0) |
| ***Tolterodine*** | 6460 (6.0) | Risperidone | 234 (0.2) | Hyoscine hydrobromide | 44 (0.0) |
| Atenolol | 4728 (4.4) | ***Trihexyphenidyl*** | 225 (0.2) | Isosorbide dinitrate | 42 (0.0) |
| Dipyridamole | 4020 (3.7) | ***Hydroxyzine*** | 219 (0.2) | Levocetirizine | 33 (0.0) |
| Diazepam | 3854 (3.6) | ***Promethazine*** | 209 (0.2) | Pethidine | 29 (0.0) |
| Isosorbide mononitrate | 3607 (3.3) | Desloratadine | 188 (0.2) | ***Trimipramine*** | 26 (0.0) |
| Carbamazepine | 2947 (2.7) | Theophylline | 185 (0.2) | Hydrocortisone sodium succinate | 25 (0.0) |
| Digoxin | 2733 (2.5) | **I*mipramine*** | 157 (0.1) | Peru Balsam / zinc oxide / pramocaine / benzyl benzoate / bismuth oxide / bismuth subgallate / hydrocortisone acetate | 25 (0.0) |
| Morphine | 2633 (2.4) | Nefopam | 145 (0.1) | Simeticone / dicycloverine / magnesium oxide light / aluminium hydroxide dried | 24 (0.0) |
| Venlafaxine | 2567 (2.4) | ***Chlorpromazine*** | 143 (0.1) | Levomepromazine hydrochloride | 23 (0.0) |
| Ranitidine | 2496 (2.3) | Captopril | 138 (0.1) | Benzyl benzoate / bismuth oxide / bismuth subgallate / hydrocortisone acetate / Peru Balsam / zinc oxide | 18 (0.0) |
| ***Trospium*** | 1887 (1.7) | ***Clomipramine*** | 135 (0.1) | ***Trifluoperazine*** | 15 (0.0) |
| Amantadine | 1799 (1.6) | Aripiprazole | 131 (0.1) | Hydrocortisone acetate | 13 (0.0) |
| Codeine phosphate | 1638 (1.5) | Diphenoxylate / ***atropine*** | 125 (0.1) | Aspirin / isosorbide mononitrate | 12 (0.0) |
| Nifedipine | 1551 (1.4) | ***Disopyramide*** | 106 (0.1) | Pseudoephedrine / ***brompheniramine*** | 8 (0.0) |
| Loperamide | 1435 (1.3) | Colchicine | 103 (0.1) | Bupropion | 6 (0.0) |
| Cetirizine | 1297 (1.2) | ***Darifenacin*** | 100 (0.1) | ***Doxepin*** | 6 (0.0) |
| Dipyridamole / aspirin | 1091 (1.0) | Cimetidine | 99 (0.1) | ***Benztropine*** | 5 (0.0) |
| Fentanyl | 1055 (1.0) | Levomepromazine maleate | 95 (0.1) | Diphenhydramine | 2 (0.0) |
| ***Fesoterodine*** | 976 (0.9) | Atenolol / chlortalidone | 92 (0.1) | Alprazolam | 1 (0.0) |
| Loratadine | 903 (0.8) | ***Orphenadrine*** | 91 (0.1) | ***Atropine*** | 1 (0.0) |
| ***Paroxetine*** | 847 (0.8) | Hydrocortisone | 84 (0.1) | ***Clemastine*** | 1 (0.0) |
| Quetiapine | 841 (0.8) | ***Propantheline*** | 81 (0.1) | ***Clozapine*** | 1 (0.0) |
| Hyoscine butylbromide | 673 (0.6) | Cinchocaine / hydrocortisone | 69 (0.1) | Hydrocortisone sodium phosphate | 1 (0.0) |
| Trazodone | 665 (0.6) | Hydralazine | 68 (0.1) | Ibuprofen / codeine | 1 (0.0) |
| Metoprolol | 488 (0.5) | ***Amitriptyline / perphenazine*** | 60 (0.1) | Morphine / cyclizine | 1 (0.0) |
| Amiloride / furosemide | 454 (0.4) | ***Solifenacin*** / tamsulosin | 60 (0.1) |  |  |

Bold-italics text = drugs with strong anticholinergic properties (American Geriatrics Society 2015 Beers Criteria Update Expert Panel)^7^

TABLE S5 OAB drug combinations during the 12-month follow-up period (overall study cohort)

| **Drug 1** | **Drug 2** | ***N* (%)^a^** |
| --- | --- | --- |
| Solifenacin | Tolterodine | 58 (14.7) |
| Oxybutynin IR | Tolterodine | 56 (14.2) |
| Oxybutynin IR | Solifenacin | 47 (11.9) |
| Oxybutynin ER | Oxybutynin IR | 33 (8.4) |
| Tolterodine | Trospium | 25 (6.3) |
| Solifenacin | Trospium | 20 (5.1) |
| Oxybutynin ER | Tolterodine | 19 (4.8) |
| Oxybutynin IR | Trospium | 18 (4.6) |
| Oxybutynin ER | Solifenacin | 18 (4.6) |
| Fesoterodine | Solifenacin | 12 (3.0) |
| Mirabegron | Solifenacin | 11 (2.8) |
| Flavoxate | Oxybutynin IR | 10 (2.5) |
| Propiverine | Tolterodine | 9 (2.3) |
| Fesoterodine | Tolterodine | 7 (1.8) |
| Flavoxate | Solifenacin | 6 (1.5) |
| Fesoterodine | Trospium | 5 (1.3) |
| Fesoterodine | Oxybutynin IR | 5 (1.3) |
| Mirabegron | Oxybutynin IR | 5 (1.3) |
| Propiverine | Trospium | 4 (1.0) |
| Mirabegron | Trospium | 3 (0.8) |
| Flavoxate | Tolterodine | 3 (0.8) |
| Oxybutynin IR | Propiverine | 2 (0.5) |
| Oxybutynin ER | Trospium | 2 (0.5) |
| Mirabegron | Tolterodine | 2 (0.5) |
| Mirabegron | Oxybutynin ER | 2 (0.5) |
| Propiverine | Solifenacin | 2 (0.5) |
| Darifenacin | Oxybutynin IR | 2 (0.5) |
| Darifenacin | Mirabegron | 2 (0.5) |
| Fesoterodine | Oxybutynin ER | 1 (0.3) |
| Fesoterodine | Mirabegron | 1 (0.3) |
| Flavoxate | Trospium | 1 (0.3) |
| Flavoxate | Oxybutynin ER | 1 (0.3) |
| Darifenacin | Solifenacin | 1 (0.3) |
| Darifenacin | Trospium | 1 (0.3) |

ER, extended release; IR, immediate release; OAB, overactive bladder.

^a^Some patients were prescribed multiple combinations.

TABLE S6 Drug utilisation during the 12-month follow-up period by age and gender

| **Variable** | | **19–65 years**  **(*N*=2038)** | **>65 years**  **(*N*=1875)** | **Male**  **(*N*=2334)** | **Female**  **(*N*=1579)** |
| --- | --- | --- | --- | --- | --- |
| Number of oral OAB drug prescriptions | Mean (SD) | 6.9 (8.1) | 7.0 (8.3) | 6.9 (8.2) | 7.0 (8.1) |
| Number of oral OAB drug prescriptions, *n* (%) | 0 | 243 (11.9) | 64 (3.4) | 181 (7.8) | 126 (8.0) |
|  | 1–4 | 724 (35.5) | 847 (45.2) | 944 (40.4) | 627 (39.7) |
|  | 5–9 | 483 (23.7) | 428 (22.8) | 540 (23.1) | 371 (23.5) |
|  | 10–14 | 441 (21.6) | 400 (21.3) | 508 (21.8) | 333 (21.1) |
|  | 15–44 | 118 (5.8) | 113 (6.0) | 128 (5.5) | 103 (6.5) |
|  | ≥45 | 29 (1.4) | 23 (1.2) | 33 (1.4) | 19 (1.2) |
| Cumulative numbers of days’ supply of oral OAB drugs | Mean (SD) | 208 (247) | 197 (163) | 201 (179) | 206 (251) |
| Cumulative numbers of days’ supply of oral OAB drugs, *n* (%) | 0–29 | 463 (22.7) | 341 (18.2) | 476 (20.4) | 328 (20.8) |
|  | 30–119 | 439 (21.5) | 513 (27.4) | 579 (24.8) | 373 (23.6) |
|  | 120–349 | 568 (27.9) | 539 (28.7) | 641 (27.5) | 466 (29.5) |
|  | 350–549 | 538 (26.4) | 465 (24.8) | 615 (26.3) | 388 (24.6) |
|  | ≥550 | 30 (1.5) | 17 (0.9) | 23 (1.0) | 24 (1.5) |
| Oral OAB drug combination use, *n* (%) | Yes | 166 (8.1) | 146 (7.8) | 147 (6.3) | 165 (10.4) |
| Number of prescriptions for antibiotics for UTI^a^ | Mean (SD) | 2.3 (4.2) | 2.1 (3.9) | 1.7 (3.5) | 2.9 (4.6) |
| Number of prescriptions for antibiotics for UTI^a^, *n* (%) | 0 | 954 (46.8) | 849 (45.3) | 1221 (52.3) | 582 (36.9) |
|  | 1–4 | 753 (36.9) | 767 (40.9) | 855 (36.6) | 665 (42.1) |
|  | 5–9 | 199 (9.8) | 153 (8.2) | 158 (6.8) | 194 (12.3) |
|  | 10–14 | 87 (4.3) | 68 (3.6) | 62 (2.7) | 93 (5.9) |
|  | 15–19 | 44 (2.2) | 37 (2.0) | 37 (1.6) | 44 (2.8) |
|  | ≥20 | 1 (0.0) | 1 (0.1) | 1 (0.0) | 1 (0.1) |
| Number of α-adrenergic antagonist or 5-ARI prescriptions^b^ | Mean (SD) | 1.5 (5.5) | 4.5 (9.7) | 4.7 (9.7) | 0.3 (2.4) |
| Number of α-adrenergic antagonist or 5-ARI prescriptions,^b^ *n* (%) | 0 | 1743 (85.5) | 1173 (62.6) | 1384 (59.3) | 1532 (97.0) |
|  | 1–4 | 75 (3.7) | 148 (7.9) | 216 (9.3) | 7 (0.4) |
|  | 5–9 | 97 (4.8) | 199 (10.6) | 277 (11.9) | 19 (1.2) |
|  | 10–14 | 87 (4.3) | 213 (11.4) | 283 (12.1) | 17 (1.1) |
|  | 15–19 | 29 (1.4) | 122 (6.5) | 149 (6.4) | 2 (0.1) |
|  | ≥20 | 7 (0.3) | 20 (1.1) | 25 (1.1) | (0.1) |

5-ARI, 5α-reductase inhibitors; OAB, overactive bladder; SD, standard deviation; UTI, urinary tract infection.
^a^Trimethoprim, ciprofloxacin, nitrofurantoin, amoxicillin, amoxicillin/clavulanic acid at any dosage.
^b^Doxazosin, tamsulosin, alfuzosin, terazosin, finasteride, dutasteride at any dosage.

TABLE S7 Complications during the 12-month follow-up period by age and gender

| **Complication,^a^ *n* (%)** | **19–65 years**  **(*N*=2038)** | **>65 years**  **(*N*=1875)** |  | **Male**  **(*N*=2334)** | **Female**  **(*N*=1579)** |
| --- | --- | --- | --- | --- | --- |
| Urinary tract infection | 312 (15.3) | 246 (13.1) |  | 257 (11.0) | 301 (19.1) |
| Urinary incontinence | 278 (13.6) | 279 (14.9) |  | 242 (10.4) | 315 (19.9) |
| Urinary retention | 36 (1.8) | 60 (3.2) |  | 83 (3.6) | 13 (0.8) |
| Sepsis/septicaemia | 13 (0.6) | 21 (1.1) |  | 25 (1.1) | 9 (0.6) |
| Renal failure (acute or other) | 15 (0.7) | 12 (0.6) |  | 19 (0.8) | 8 (0.5) |
| Hydronephrosis | 9 (0.4) | 5 (0.3) |  | 7 (0.3) | 7 (0.4) |
| Obstructive uropathy | 1 (0.0) | 0 |  | 1 (0.0) | 0 |

^a^Each complication was identified from medical records using prespecified read codes.

TABLE S8 Resource use and costs during the 12-month follow-up period by age and gender

| **Resource** | **Variable** |  | **19–65 years**  **(*N*=2038)** | **>65 years**  **(*N*=1875)** | **Male**  **(*N*=2334)** | **Female**  **(*N*=1579)** |
| --- | --- | --- | --- | --- | --- | --- |
| GP consultations  (all-cause)^a^ | ≥1 visit | *n* (%) | 2038 (100.0) | 1875 (100.0) | 2334 (100.0) | 1579 (100.0) |
|  | Visits, *n* | Mean (SD) | 63.0 (43.2) | 72.9 (41.4) | 67.4 (42.3) | 68.3 (43.1) |
|  | Cost, £ | Mean (SD) | 1348 (970) | 1557 (952) | 1435 (959) | 1469 (978) |
|  |  | Median (range) | 1145 (37–10776) | 1366 (46–7424) | 1240 (37–10776) | 1255 (37–9382) |
| Specialist visits (urologist/ gynaecologist) | ≥1 visit (overall) | *n* (%) | 940 (46.1) | 888 (47.4) | 1146 (49.1) | 682 (43.2) |
|  | Urologist |  | 866 (42.5) | 863 (46.0) | 1144 (49.0) | 585 (37.0) |
|  | Gynaecologist |  | 122 (6.0) | 53 (2.8) | 6 (0.3) | 169 (10.7) |
|  | Visits, *n* (overall) | Mean (SD) | 2.2 (1.6) | 2.3 (1.7) | 2.3 (1.7) | 2.2 (1.5) |
|  | Urologist |  | 2.2 (1.6) | 2.2 (1.6) | 2.3 (1.7) | 2.0 (1.4) |
|  | Gynaecologist |  | 1.7 (1.1) | 2.3 (1.7) | 1.2 (0.4) | 1.9 (1.4) |
|  | Cost, £ (overall) | Mean (SD) | 251 (180) | 254 (193) | 253 (187) | 252 (186) |
|  |  | Median (range) | 219 (109–1641) | 219 (109–1312) | 219 (109–1641) | 219 (109–1203) |
| Incontinence pads | ≥1 pad | *n* (%) | 9 (0.4) | 5 (0.3) | 3 (0.1) | 11 (0.7) |
|  | Prescriptions, *n* | Mean (SD) | 6.0 (6.8) | 3.8 (4.2) | 1.0 (0.0) | 6.4 (6.3) |
|  | Cost, £ | Mean (SD) | 57 (52) | 11 (8) | 9 (2) | 49 (50) |
|  |  | Median (range) | 50 (6–126) | 6 (4–24) | 10 (6–10) | 24 (4–126) |
| Urodynamics | ≥1 test | *n* (%) | 48 (2.4) | 50 (2.7) | 51 (2.2) | 47 (3.0) |
|  | Tests, *n* | Mean (SD) | 1.5 (0.9) | 1.3 (0.6) | 1.2 (0.4) | 1.7 (0.9) |
|  | Cost, £ | Mean (SD) | 192 (113) | 166 (69) | 148 (49) | 212 (118) |
|  |  | Median (range) | 126 (126–630) | 126 (126–378) | 126 (126–252) | 126 (126–630) |
| Cytoscopy | ≥1 test | *n* (%) | 154 (7.6) | 189 (10.1) | 210 (9.0) | 133 (8.4) |
|  | Tests, *n* | Mean (SD) | 1.1 (0.4) | 1.2 (0.5) | 1.2 (0.5) | 1.1 (0.3) |
|  | Cost, £ | Mean (SD) | 166 (63) | 175 (69) | 177 (75) | 161 (48) |
|  |  | Median (range) | 146 (146–438) | 146 (146–584) | 146 (146–584) | 146 (146–438) |
| Imaging^b^ | ≥1 test | *n* (%) | 49 (2.4) | 34 (1.8) | 45 (1.9) | 38 (2.4) |
|  | Tests, *n* | Mean (SD) | 1.1 (0.3) | 1.1 (0.2) | 1.1 (0.3) | 1.1 (0.2) |
|  | Cost, £ | Mean (SD) | 110 (85) | 88 (80) | 87 (93) | 117 (66) |
|  |  | Median (range) | 144 (0–288) | 144 (0–288) | 83 (0–288) | 144 (0–288) |
| Procedures and surgical interventions (urology)^c,d^ | ≥1 procedure | *n* (%) | 121 (5.9) | 102 (5.4) | 145 (6.2) | 78 (4.9) |
|  | Procedures, *n* | Mean (SD) | 1.3 (0.6) | 1.4 (0.7) | 1.4 (0.72) | 1.3 (0.6) |
|  | Cost, £ | Mean (SD) | 1966 (2592) | 2679 (5092) | 2392 (4510) | 2087 (2503) |
|  |  | Median (range) | 1067 (168–14934) | 1418 (220–47419) | 1074 (168–47419) | 1129 (168–14934) |
| Hospitalisations (urology)^d,e^ | ≥1 hospitalisation | *n* (%) | 207 (10.2) | 224 (11.9) | 262 (11.2) | 169 (10.7) |
|  | Hospitalisations, *n* | Mean (SD) | 1.8 (1.6) | 1.4 (0.9) | 1.6 (1.3) | 1.6 (1.2) |
|  | Admitted days, *n* | Mean (SD) | 12.9 (32.0) | 12.1 (20.5) | 12.9 (28.1) | 11.9 (24.1) |
|  | Cost, £ | Mean (SD) | 6687 (15588) | 5859 (11193) | 6880 (15605) | 5284 (9172) |
|  |  | Median (range) | 2191 (162–163720) | 2952 (162–134885) | 2798 (162–163720) | 2257 (162–80325) |
| Total | Cost, £ | Mean (SD) | 2269 (5804) | 2532 (4951) | 2488 (6200) | 2257 (3972) |
|  |  | Median (range) | 1340 (37–166644) | 1578 (46–137794) | 1456 (37–166644) | 1462 (37– 90610) |

GP, general practitioner; MS, multiple sclerosis; PD, Parkinson’s disease; SB, spina bifida; SCI, spinal cord injury; SD, standard deviation; STK, stroke.

^a^Includes surgery and clinical consultations, home visits, out-of-hours visits and telephone consultations.

^b^Cystography, ultrasound, computed tomography, x-ray, magnetic resonance imaging, other diagnostic imaging of bladder, spine, genitourinary system, pelvis or abdomen.

^c^Includes intermittent catheterisation, indwelling catheterisation, botulinum toxin A injections, sacral nerve stimulation, bladder augmentation, sling procedures, and artificial urinary sphincter.

^d^Derived from the Hospital Episode Statistics (HES) database. Patients without linked data available from HES were assumed to have not utilised these resources. Numbers of hospital admissions or procedures and surgical interventions were calculated among patients who had ≥1 resource use item.

^e^Admissions related to a urological disease, or to PD, MS, SCI, STK or SB with a related urological disease. Urological diseases included pyelonephritis, sepsis, hydronephrosis, uropathy, renal failure, chronic kidney disease, calculus, cystitis, neuropathic bladder, urethritis, urinary tract infection, proteinuria, incontinence, and retention.

FIGURE S1 Patient selection criteria

**Patients with definitive NGB**


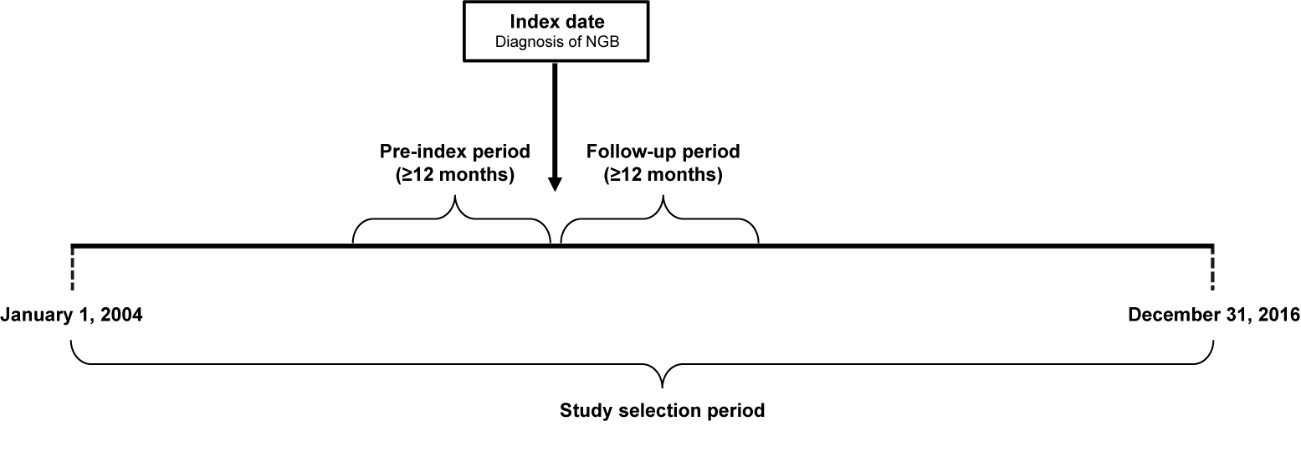


**Patients with probable NGB (PD, MS, SCI or stroke)**


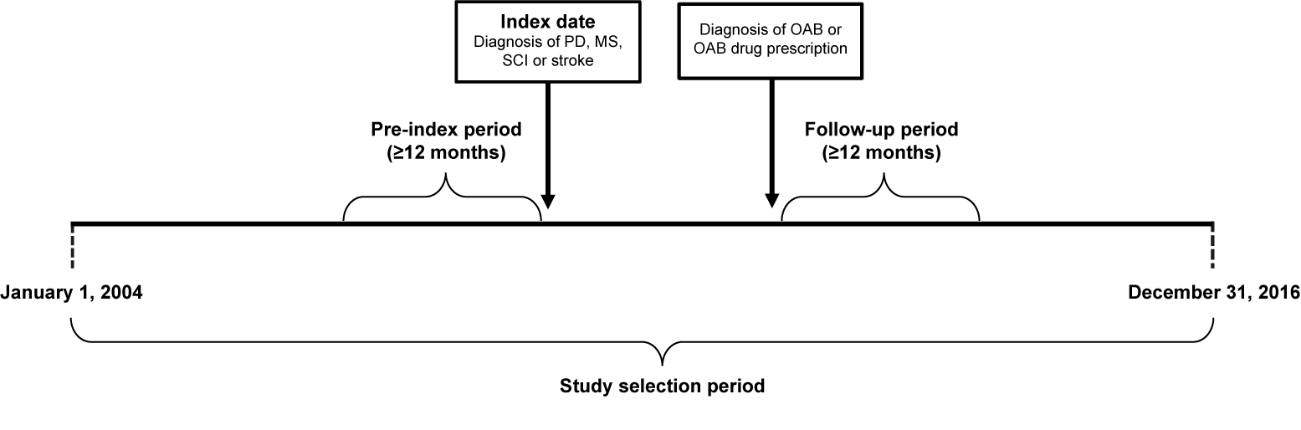


**Patients with probably NGB (spina bifida)**

**
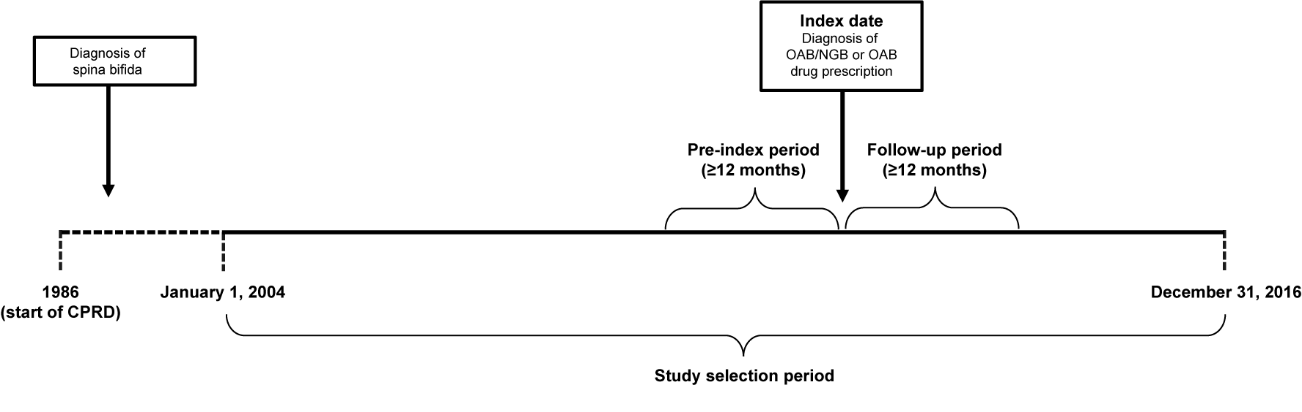
**

Note: Periods are not drawn to scale.

CPRD, Clinical Practice Research Datalink; MS, multiple sclerosis; NGB, neurogenic bladder; OAB, overactive bladder; PD, Parkinson’s disease; SCI, spinal cord injury.

**REFERENCES**

1. Boustani M, Campbell N, Munger S, Maidment I, Fox C. Impact of anticholinergics on the aging brain: a review and practical application. *Aging Health*. 2008;4(3):311–320.
2. Aging Brain Care. Anticholinergic Cognitive Burden Scale, 2012 update. Available at: [www.agingbraincare.org/uploads/products/ACB_scale_-_legal_size.pdf](http://www.agingbraincare.org/uploads/products/ACB_scale_-_legal_size.pdf). Accessed October 2018.
3. Curtis L, Burns A. Personal Social Services Research Unit (PSSRU). Unit costs of health and social care 2017. Available at: <https://www.pssru.ac.uk/project-pages/unit-costs/unit-costs-2017/>. Accessed October 2018.
4. Curtis L. Personal Social Services Research Unit (PSSRU). Unit costs of health and social care 2017. Available at: <https://www.pssru.ac.uk/project-pages/unit-costs/unit-costs-2014/>. Accessed October 2018.
5. National Health Service (NHS) National schedule of reference costs 2016-2017. Available at: <https://improvement.nhs.uk/resources/reference-costs/>. Accessed October 2018.
6. Age UK Incontinence. Lille Healthcare Classic Pad PE Backed Maxi (1250 ml) 30 pack. Available at: [https://www.ageukincontinence.co.uk/lille-healthcare-classic-pad-maxi-plus-insert-pads-1250ml-pack-of-30.html. Accessed October 2018](https://www.ageukincontinence.co.uk/lille-healthcare-classic-pad-maxi-plus-insert-pads-1250ml-pack-of-30.html.%20Accessed%20October%202018).
7. American Geriatrics Society 2015 Beers Criteria Update Expert Panel. American Geriatrics Society 2015 Updated Beers Criteria for Potentially Inappropriate Medication Use in Older Adults. J Am Geriatr Soc 2015;63(11):2227–46.
